# Therapeutic effect of *Yang-Xue-Qing-Nao* granules on sleep dysfunction in Parkinson’s disease

**DOI:** 10.1186/1749-8546-8-14

**Published:** 2013-07-27

**Authors:** Weidong Pan, Shin Kwak, Guoyan Li, Yiyun Chen, Dingfang Cai

**Affiliations:** 1 Department of Neurology, Shuguang Hospital Affiliated to Shanghai University of Traditional Chinese Medicine, 185, Pu-An Road, Shanghai 200021, China; 2 Department of Neurology, Graduate School of Medicine, The University of Tokyo, 7-3-1, Hongo, Bunkyo-ku, Tokyo 113-8655, Japan; 3 Japan Judo Therapist Association, Chinese-Japanese Osteopaths Court, 4-44-23, Honchio, Nakano-ku, Tokyo 164-0012, Japan; 4 Laboratory of Neurology, Institute of Integrative Medicine, Zhongshan Hospital, Fudan University, 180, Fenglin Road, Shanghai 200032, China

## Abstract

**Background:**

This study aimed to evaluate the effects of add-on *Yang-Xue-Qing-Nao* granules (YXQN) on sleep dysfunction in patients with Parkinson’s disease (PD).

**Methods:**

PD participants fitted with an actigraph took either YXQN or placebo granules in a randomized manner for 12 weeks while maintaining other anti-parkinsonism medications (*e.g.*, dopaminergic agent, dopamine agonist) unchanged. Additional participants without sleep disturbance or PD served as controls. The changes in detrended fluctuation analysis (DFA) of physical activity with respect to diurnal activity (DA), evening activity (EA), nocturnal activity (NA), Parkinson’s disease sleep scale (PDSS) score and unified Parkinson’s disease rating scale (UPDRS) score were evaluated every 4 weeks during the 12-week YXQN intervention period and again at week 16.

**Results:**

A total of 61 (placebo group, n = 30; YXQN group, n = 31) idiopathic PD participants with sleep dysfunction (mean age ± standard deviation, 63.4 ± 8.6 years; mean duration of illness, 5.8 ± 6.6 years) completed the study. Significant improvements in EA (p = 0.033, 0.037 and 0.029), DA (p = 0.041, 0.038 and 0.027) and PDSS score (p = 0.034, 0.028 and 0.029) were observed in the YXQN group at weeks 8 and 12, and maintained until week 16, respectively.

**Conclusion:**

YXQN improved the DFA parameters, and PDSS and UPDRS scores in PD participants.

## Introduction

A high incidence of sleep dysfunction has been reported in patients with Parkinson’s disease (PD) [1-3], especially in patients receiving dopaminergic agent treatment [3,4]. PD patients with significant sleep dysfunction may indicate early progressive degeneration of the sleep centers [5]. Chinese medicine (CM) preparations have shown beneficial effects on aging-related symptoms [6,7] and chronic diseases, such as PD [8-13], including improved nocturnal sleep disorder in PD patients [14].

As a CM compound for treating headache and dizziness associated with serious sleep disturbances, *Yang-Xue-Qin-Nao* granules (YXQN) attenuate cerebral microcirculatory disturbances during ischemia-reperfusion injury *in vivo*[15], which might improve the sleep dysfunction in PD patients. YXQN (Tianjin Tasly Pharmaceutical Co. Ltd., Tianjin, China) is a newly developed compound medicine that was approved in 1996 by the China State Food and Drug Administration for the treatment of headache and dizziness associated with cerebrovascular diseases. The granules are composed of 11 herbs: Radix angelicae sinensis (*Dang Gui*), 6.76%; Rhizoma chuanxiong (*Chuan Xiong*), 6.76%; Radix paeoniae alba (*bai shao*), 5.41%; Ramulus uncariae cum uncis (*Gou Teng*), 13.51%; Caulis spatholobi (*Ji Xue Teng*), 13.51%; Spica prunellae (*Xia Ku Cao*), 13.51%; Concha margaritifera usta (*Zhen Zhu Mu*), 13.51%; Radix rehmanniae preparata (*Di Huang*), 5.41%; Semen cassiae (*Jue Ming Zi*), 13.51%; Rhizoma corydalis yanhusuo (*Yan Hu Suo*), 6.76%; and Herba asari (*Xi Xin*), 1.35%. Available evidence from *in vitro* studies has revealed that at least six chemicals derived from these herbs exhibit antioxidant potential, inhibit neutrophil adhesion to endothelial cells and decrease infarct size and brain tissue damage [15]. YXQN was reported to show effects on functional and structural changes in microvessels in the cerebral cortex of gerbils induced by ischemia-reperfusion injury [15]. These granules might improve non-motor symptoms, such as dizziness, headache and even depression [16,17].

This study aimed to evaluate the effects of add-on YXQN in PD patients by analyzing actigraphic measurements [18,19]. The changes in detrended fluctuation analysis (DFA) parameters were analyzed by the actigraph records for diurnal activity (DA), evening activity (EA) and nocturnal activity (NA).

## Methods

### Participant inclusion and exclusion criteria

The UK Parkinson’s Disease Society Brain Bank clinical diagnostic criteria were used to assess the eligibility for participation in this study. PD was defined by the presence of at least two of the four cardinal features (bradykinesia, tremor, rigidity and postural reflex abnormality). Subjects were included if they had idiopathic PD, were being treated with daytime levodopa and had at least three awakenings per night occurring at least three nights per week and attributable to PD symptoms [20]. Exclusion criteria were current direct dopamine agonist treatment, dementia, depression, psychosis, hallucinations, and prior intolerance to dopamine agonists or use of sleep medications. All patients were at least 40 years of age, were evaluated in the middle of their levodopa dose cycle at maximal mobility (‘on’) for the severity of parkinsonism and signed informed consent before participation. The study was approved by The Ethics Committee of Shuguang Hospital Affiliated to Shanghai University of Traditional Chinese Medicine, and was performed in accordance with the principles outlined in the Declaration of Helsinki.

### Randomization, masking and drug administration

An unmasked pharmacist generated randomization codes using an Excel (Microsoft Office) random number generator (Microsoft, USA) in blocks of two and four participants. Kits were given sequential numbers that corresponded to the randomization key and were maintained in a secure location. When randomized, each successive participant was assigned by an electronic Clinical Trial Management System to the next numbered kit in sequence at each site. YXQN and the placebo granules (one-tenth active compound of YXQN with a flavor enhancer) were manufactured by the same pharmaceutical company (Tianjin Tasly Pharmaceutical Co. Ltd., China), and the shape and color of both types of granules were identical (non-distinguishable by the participants). Tablets containing YXQN (4 g) or placebo granules (4 g) were packaged into kits of several blister packs (1 week of treatment per pack). All participants and study personnel were blinded to the treatment assignment. The PD participants were randomized into either a YXQN group or a placebo group, and were required to take the granules three times per day for 12 consecutive weeks. Anti-parkinsonian drug administration was not changed throughout the study (Table 1). The participants were not treated by any other complementary and alternative treatments, such as other CM, *Tai Chi* exercise or acupuncture. The control group consisted of participants with one or more conditions such as idiopathic hypertension, chronic nephritis, chronic inflammatory demyelinating polyneuropathy and coronary heart disease, but no sleep disturbance or PD, who were diagnosed by physicians and neurologists. The control participants were invited to participate in a 1-week consecutive recording session to evaluate their sleep characteristics. Their activities and DFA parameters were recorded for comparison, because we found that the scores of the healthy controls changed only very slightly after 1 year (Weidong Pan, unpublished observation).

**Table 1:** Subject characteristics

**Subjects**	**Control group (n = 35)**	**Placebo group (n = 30)**	**YXQN group (n = 31)**	***P *value**
Age (yr)	66.8 ± 7.7	67.1 ± 10.2	68.6 ± 9.2	0.89
Men/Women	21/14	19/11	18/13	-
Disease duration	-	6.1 ± 4.9	5.9 ± 4.7	0.17
UPDRS score	-	37.59 ± 10.51	38.35 ± 6.78	0.23
Levodopa/DCI (mg/day)	-	412.5 ± 162.8	421.3 ± 142.9	0.65
Pramipexole(mg/day)	-	1.11 ± 0.39	1.03 ± 0.78	0.08
Entacapone (mg/day)	-	198.28 ± 88.47	203.67 ± 78.38	0.12
Selegiline hydrochloride (mg/day)	-	8.33 ± 6.94	8.21 ± 3.76	0.26
Adamantatamine (mg/day)	-	118.73 ± 96.59	116.31 ± 89.6	0.19
Artane hydrochloride (mg/day)	-	4.82 ± 2.91	4.65 ± 2.36	0.08

### Equipment and data analysis

Each participant wore an actigraph which was a small watch-type activity monitor equipped with a computer (*MicroMini-Motionlogger*; Ambulatory Monitoring Inc., USA), on the wrist of their non-dominant hand for 6 consecutive days in the series time windows (4 weeks each) during the 16-week observation period. Zero-crossing counts were recorded every minute by the actigraph to quantify the level of physical activity. The data were transmitted to a computer and the activity scores were plotted for 3 consecutive days to determine the daily profiles and biological rhythms of each participant. The data acquired from the actigraph were categorized into DA (between 6 a.m. and 6 p.m.), EA (between 6 p.m. and 9 p.m.) and NA (between 9 p.m. and 6 a.m.). Discontinuous data were combined through an integrative method as follows. We divided the difference between the two connecting terminals of the two data into four quarters, made the connection into an oblique line (it was a broken line before integration) to avoid connecting errors, and connected all the pieces of data into one continuous sequence for fluctuation analysis [18]. Subsequently, we analyzed the DFA value α to evaluate the relationships between time scales and magnitudes of fluctuation (standard deviations: SDs) within each time scale [21,22]. More correlated signals represented a greater growth of the fluctuation magnitude with increasing time scale or length of data windows [18]. The outcome measures were assessed at baseline and every 4 weeks during the 16-week intervention.

### Clinical evaluation

The unified Parkinson’s disease rating scale (UPDRS) score [23] and Parkinson's disease sleep scale (PDSS) score [24] were evaluated for all participants at week 0 (baseline) and at weeks 4, 8, 12 and 16 by neurologists who were blinded to the test granules. The PDSS score in the control group was evaluated once on the first day of the recording week.

### Statistical analysis

Repeated-measure ANOVAs were conducted to test the differences among changes in outcomes at baseline and every 4 weeks for 16 weeks. When a significant difference was detected, a *post hoc* test (Bonferroni test) was conducted between the YXQN group and the placebo group to compare the UPDRS total scores, the α values of DA, EA and NA, and the changes in the PDSS scores. The differences at baseline between the control group and the YXQN group, and between the control group and the placebo group were analyzed by *t*-tests. A significant difference was defined as *P* < 0.05. SPSS for Windows version 17.0 (SPSS China, China) was used for statistical analyses. All data were expressed as the mean ± SD.

## Results

Eighty-two idiopathic PD patients at the Department of Neurology of Shuguang Hospital Affiliated to Shanghai University of Traditional Chinese Medicine were recruited, and 70 patients who met the criteria were enrolled. Five PD participants in the YXQN group withdrew from the study for drug-related reasons: two participants experienced nausea and three were unable to tolerate the bitter taste of YXQN. Four participants in the placebo group dropped out: one participant was unable to tolerate the bitter taste of placebo, two participants dropped out because of inefficacy and one dropped out through a conflict with other CM preparations prescribed for concomitant diseases.

A total of 61 PD participants completed the trial, and were randomized into the YXQN group (n = 31) and the placebo group (n = 30) (Table 1). All control participants (n = 35) completed 1 week of recording. No differences in age or sex distribution were observed among the three groups. No differences in PD duration, UPDRS total score, PDSS score, α values of DFA for DA, EA and NA or dose of levodopa at baseline were observed between the YXQN group and the placebo group (Table 1).

**Figure 1:**
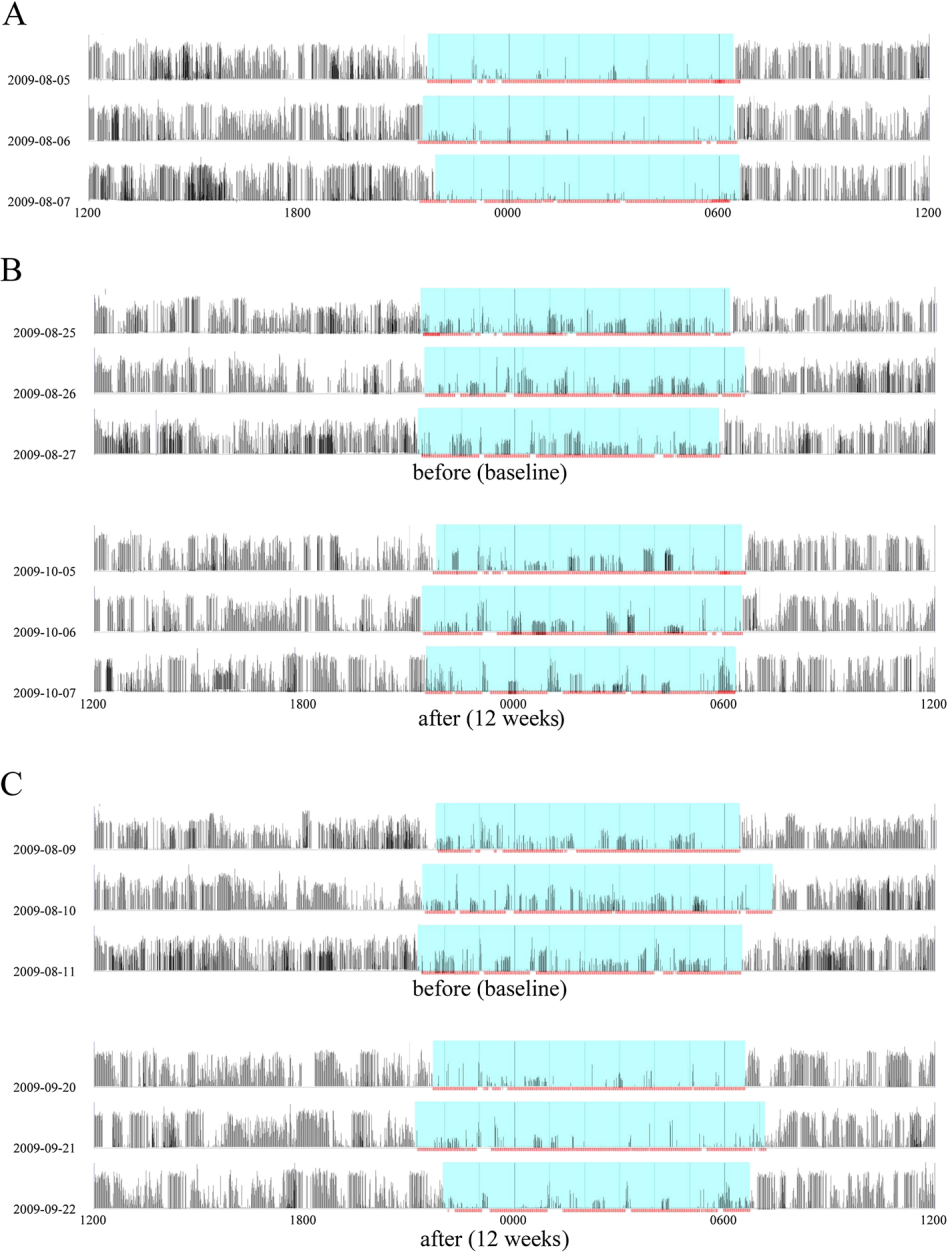
**Daily profiles of actigraph counts showing the biological rhythm before and after granule ingestion in the control group (A), placebo group (B), and *Yang-Xue Qing-Nao *granule (YXQN) group (C). **The light green color indicates sleep duration.

**Figure 2:**
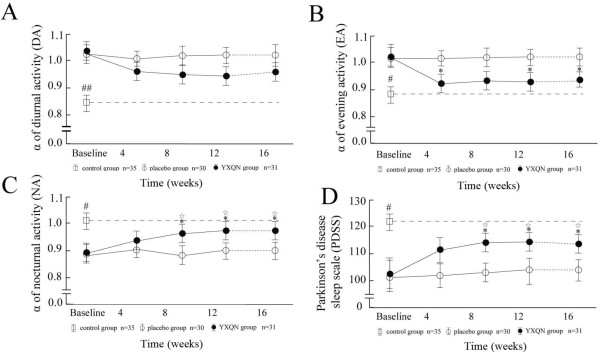
**Mean changes in *α *values of detrended fluctuation analysis (DFA) in diurnal activity (DA) value (A), evening activity (EA) value (B), diurnal activity (DA) value (C) and Parkinson’s disease sleep scale (PDSS) score (D) at baseline, and at weeks 4, 8, 12 and 16.** Black circles indicate the *Yang-Xue Qing-Nao *granule (YXQN) group, white circles indicate the placebo group and white squares indicate the control group. *P < 0.05, *vs. *baseline; ^☆^*P *< 0.05, *vs. *placebo group; ^#^*P *< 0.05, ^##^*P *< 0.01, *vs. *YXQN group or placebo group.

NA was decreased after the additional treatment in the YXQN group (Figure 1B and C). The improvements in NA in the YXQN group after continuous daily treatment for 12 weeks were similar to those in the control group (Figure 1A and C). In both the YXQN group and the placebo group, the α values of DFA for DA, EA and NA, and the PDSS score at baseline were significantly different from those in the control group (Figure 2). The α values of DA in the YXQN group decreased slightly during the 16-week observation period (Figure 2A). The α values for EA in the YXQN group were decreased at weeks 4, 12 and 16 compared with the placebo group (Figure 2B). Compared with the placebo group, the YXQN group had significantly increased α values for NA at weeks 8, 12 and 16, and increased PDSS values (Figure 2C and D). The better UPDRS scores were observed in the YXQN group at the end of the intervention compared with the placebo group, but there were no significant changes compared with baseline in either group of PD participants (Table 2). The improvements in the α values and PDSS score in the YXQN group were similar to those in the control group, and the effects in the YXQN group were maintained for 4 weeks (Figure 2). No serious adverse events were observed.

**Table 2:** Changes in detrended fluctuation analysis (DFA) values, Parkinson’s disease sleep scale (PDSS) and unified Parkinson’s disease rating scale (UPDRS)

**Variable**	**Mean changes from baseline (95%CI)**		**Between group difference (96%CI)**	
	**YXQN**	**Placebo**	**YXQN vs Placebo**	***P *value**
α value of DA				
Week 8	−0.9 [−1.1 to −0.7]	−0.2 [−0.3 to 0.1]	−0.7 [−1.0 to −0.8]	0.056
Week 12	−1.0 [−1.2 to −0.7]	−0.1 [−0.3 to 0.2]	−0.8 [−1.1 to −0.7]	0.051
Week 16	−0.9 [−1.1 to −0.6]	−0.2 [−0.2 to 0.3]	−0.9 [−1.1 to −0.7]	0.072
α value of EA				
Week 8	−1.1 [−1.4 to −0.6]	−0.1 [−0.1 to 0.2]	−1.0 [−1.3 to −0.5]	0.048
Week 12	−0.9 [−1.0 to −0.6]*	0.1 [−0.3 to 0.4]	−1.1 [−1.6 to −0.7]	0.036
Week 16	−0.9 [−1.1 to −0.7]*	−0.2 [−0.4 to 0.3]	−0.9 [−1.3 to −0.8]	0.041
α value of NA				
Week 8	1.0 [0.8 to 1.6]*	−0.3 [−0.6 to 0.4]	1.1 [0.7 to 1.5]	0.017
Week 12	1.1 [0.7 to 1.5]*	−0.1 [−0.5 to 0.5]	1.0 [0.6 to 1.4]	0.038
Week 16	0.9 [0.6 to 1.7]*	−0.2 [−0.5 to 0.4]	0.9 [0.7 to 1.6]	0.042
PDSS score				
Week 8	12.1 [7.6 to 13.7]*	2.9 [−4.1 to 9.7]	10.7 [6.9 to 16.5]	0.026
Week 12	11.9 [7.1 to 14.2]*	3.3 [−6.6 to 7.3]	9.8 [8.7 to 15.9]	0.029
Week 16	9.8 [7.0 to 14.6]*	3.2 [−4.9 to 5.1]	9.9 [7.4 to 17.6]	0.048
UPDRS score				
Week 8	−6.6 [−11.4 to −2.8]	−1.7 [−2.8 to 3.9]	−4.9 [−6.7 to −2.7]	0.076
Week 12	−5.7 [−10.8 to −1.9]	−0.9 [−1.7 to 2.1]	−5.1 [−8.3 to −2.4]	0.069
Week 16	−5.2 [−8.9 to −2.1]	2.1 [−4.2 to 6.1]	−4.6 [−7.5 to −3.1]	0.153

*: *P* < 0.05, compared with baseline.

## Discussion

Herbal treatments may influence PD symptoms and the effectiveness of dopaminergic therapy [25]. The improved NA associated with YXQN, but not with nocturnal levodopa, may be attributed to differences in the pharmacologic actions on dopamine receptor subtypes or the effects of YXQN on other neurotransmitter systems [15]. The UPDRS scores improved slightly after YXQN administration (Table 2), and the observed benefits might be caused by the sleep effects, rather than direct motor effects, in patients with PD.

Currently, complementary and alternative treatments are becoming more effective for the treatment of chronic disorders, such as dementia, sleep disorder, mood disorders, high blood pressure and anxiety [26-31]. However, there is no reliable quantitative and objective equipment to evaluate the effects. Several studies have demonstrated the utility of actigraphs [14,18-20] and their application in PD to assess disease severity, NA and circadian rest-activity rhythm with aging and Alzheimer’s disease [32], and to distinguish tremors from soma activity in PD patients [33]. Although actigraphs cannot distinguish between NA owing to wakefulness and NA caused by other nocturnal sleep disturbances, such as REM sleep behavior disorder, periodic limb movements or sleep apnea, none of the latter effects are caused or worsened by dopamine treatment. Nocturnal sleep disturbance may arise from these symptoms [2-4], and demonstration of improvements may indicate that the sleep disturbance can be treated by alternative therapies such as YXQN. The exact mechanisms of the effects of YXQN on REM sleep behavior disorder, periodic limb movements or sleep apnea remain to be determined.

In this study, administration of YXQN three times per day for 12 weeks was effective for improving EA and sleep dysfunction in PD participants, and these improvements were maintained for 16 weeks. A lower α value of DFA indicates a higher activity ability during the diurnal period, while a higher α value of DFA reflects a lower activity ability during the nocturnal period [18,21,22]. No treatment-related adverse events, such as hypotension, abnormal liver and/or renal function, diarrhea or coagulation defects, were observed in the study participants, indicating that YXQN is likely to be a safe therapy for PD patients with sleep dysfunction.

## Conclusion

YXQN improved the DFA parameters, and PDSS and UPDRS scores in PD participants.

## Abbreviations

PD: Parkinson’s disease; CM: Chinese medicine; YXQN: *Yang-Xue-Qing-Nao* granules; DA: Diurnal activity; EA: Evening activity; NA: Nocturnal activity; DFA: Detrended fluctuation analysis; UPDRS: Unified Parkinson’s disease rating scale.

## Competing interests

The authors declare that they have no competing interests.

## Authors’ contributions

WP, SK, DC and YC designed the study. WP, DC and GL performed the statistical analysis. WP, SK, DC and YC wrote the manuscript. All authors read and approved the final manuscript.
